# Incidentally discovered mesenteric paraganglia as large as a lymph node in the sigmoid mesocolon, a possible origin of mesenteric paraganglioma

**DOI:** 10.1111/pin.12939

**Published:** 2020-04-27

**Authors:** Daisuke Suzuki, Shiori Meguro, Yuki Watanabe, Toru Kawai, Takanori Kyokane, Yoichiro Aoshima, Yasunori Enomoto, Hideya Kawasaki, Haruna Yagi, Isao Kosugi, Mayu Fukushima, Satoshi Baba, Toshihide Iwashita

**Affiliations:** ^1^ Department of Diagnostic Pathology Chutoen General Medical Center Shizuoka Japan; ^2^ Department of Regenerative and Infectious Pathology Hamamatsu University School of Medicine Shizuoka Japan; ^3^ Department of Surgery Chutoen General Medical Center Shizuoka Japan; ^4^ Department of Diagnostic Pathology Hamamatsu University Hospital Shizuoka Japan

*To the Editor*

Here, we report a case of an accidentally discovered mesenteric paraganglia as large as a lymph node in the sigmoid mesocolon. Paraganglia are groups of neural crest‐derived paraneurons and are prominent in fetuses and young infants; however, after about 3 years of terrestrial life, they regress and become relatively sparse and less noticeable in adults. Paraganglia are categorized into two types, parasympathetic and sympathetic paraganglia, from which parasympathetic and sympathetic paragangliomas originate, respectively. Approximately 80–85% of sympathetic paragangliomas develop in the adrenal medulla and are called ‘pheochromocytoma’, whereas 15–20% of sympathetic paragangliomas occur from extra‐adrenal sympathetic paraganglia and are called ‘extra‐adrenal sympathetic paraganglioma’. Approximately 85% of extra‐adrenal sympathetic paragangliomas develop in the intra‐abdominal para‐aortic area of the urinary bladder.^1^ The sympathetic paraganglia of the retroperitoneum and urinary bladder have been well‐described,^2^ but paraganglia in other sites have been rarely characterized.

A 75‐year‐old man complained of abdominal pain and was admitted to our hospital. On physical examination, he had a blood pressure of 151/83 mmHg and an irregular pulse of 93 bpm, with atrial fibrillation. He had a cerebellar infarction 10 years ago. He had no family history of hereditary tumors. Laboratory studies yielded normal blood chemistry and hematology results with slight anemia (hemoglobin 11.8 g/dL, hematocrit 37%). Abdominal computed tomography revealed a solid mass in the descending colon. Subsequent colonoscopy followed by pathological examination of the biopsy specimens revealed that the mass consisted of differentiated tubular adenocarcinoma. Preoperative imaging revealed no regional lymph node or distant metastases. The pre‐surgical evaluation of this colon carcinoma was type 2, T3N0M0, Stage IIA. According to Japanese colorectal cancer surgery guidelines, D3 lymphadenectomy is the standard surgical option for Stage IIA, with or without lymph node metastasis. The patient underwent colectomy with regional lymph node resection.

Pathological examination of the resected colon cancer revealed that the differentiated tubular adenocarcinoma infiltrated the subserosal layer without lymph node metastasis. The post‐surgical evaluation was type 2, 35 × 35 mm, pT3N0M0, pStage IIA. Microscopically, among the tissues excised as lymph nodes of the sigmoid mesocolon, at least six separate nodules (diameter: 0.5–4 mm) closely gathered to form a total size of 7 × 4 mm, and each nodule appeared partially encapsulated (Fig. 1a). The nodules were virtually identical to those of the adrenal medulla (Fig. 1b). In addition, multiple tiny nests (diameter: ≤0.1 mm) were scattered around the nodules (Fig. S1a,b). Further, myelinated nerve bundles were found near the nodules (Fig. S1c).

**Figure 1:**
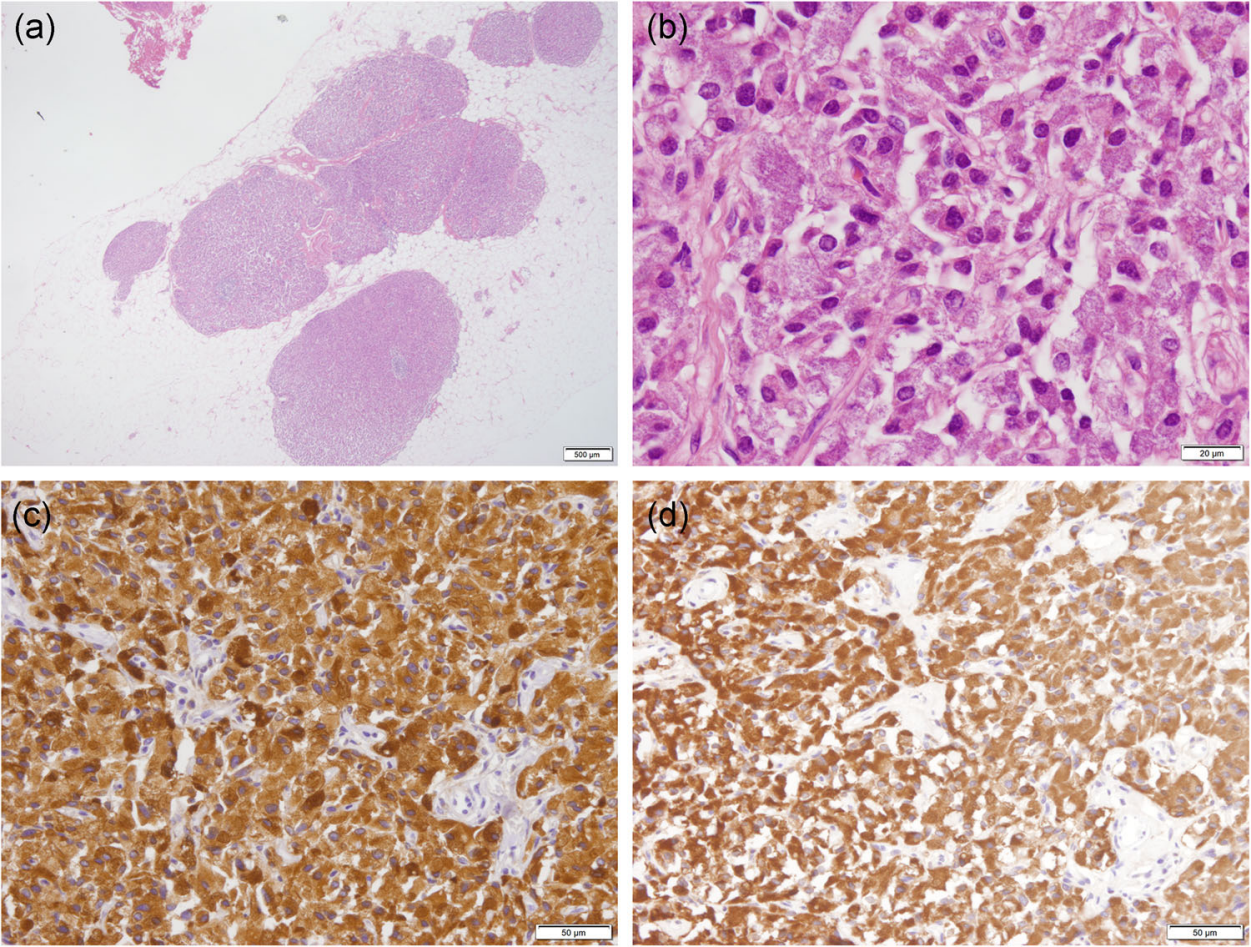
Histology and immunohistochemical staining. (**a**) A lower magnification image of the nodules after hematoxylin and eosin staining (×12.5). (**b**) A higher magnification image of the nodules after hematoxylin and eosin staining (×400). (**c**) The cells showed positive expressions of chromogranin A (×200). (**d**) The cells showed positive expressions of dopamine beta‐hydroxylase (×200). Scale bars = 500 µm (**a**), 20 µm (**b**) and 50 µm (**c** and **d**).

Immunohistochemically, most cells of all nodules and multiple tiny nests were positive for chromogranin A (Fig. 1c), CD56 (Fig. S1d,e), and synaptophysin (Fig. S1f,g), but were negative for pan‐cytokeratin marker (AE1/AE3) (Fig. S1h,i). In addition, the nodules contained scattered S100‐positive cells (Fig. S1j,k). Ki‐67 staining indicated that the proliferating cells were almost undetectable (Fig. S1l). Regarding catecholamine synthesis enzymes, tyrosine hydroxylase (Fig. S1m,n), aromatic L‐amino acid decarboxylase (Fig. S1o,p), and dopamine beta‐hydroxylase (Fig. 1d) were expressed in most of the cells. In contrast, phenylalanine N‐methyltransferase, which converts norepinephrine to epinephrine, was not expressed (Fig. S1q,r), indicating that the nodules and multiple tiny nests were sympathetic paraganglia, producing dopamine or norepinephrine, but not epinephrine.

Histological and immunohistochemical analyses showed that the nodules and multiple tiny nests were mesenteric paraganglia that persisted until adulthood. We thought that the paraganglionic tissue was non‐neoplastic because it consisted of multiple nodules with individual encapsulation, and its histology closely resembled that of the adrenal medulla. The expression levels of succinate dehydrogenase complex iron sulfur (SDH) subunit B (SDHB), which is one of the involved genes in extra‐adrenal paraganglioma was normal, indicating that the sympathetic paraganglionic tissue did not have any mutations in the *SDH* genes including *SDH subunit A, SDHB, SDH subunit C*, and *SDH subunit D* (Fig. S1s,t). The antibodies used in this study are detailed in the Table S1.

Paragangliomas arising from unusual sites have been reported to occur in the liver, orbit, mandible, paranasal sinuses and sellar region, thyroid gland, parathyroid, mediastinum, lung, heart, gut, pancreas and the mesentery (Supporting References). More than 20 cases of mesenteric paraganglioma have been reported in the English literature since 1966.^3^ However, to date, only one report has tried to explain the origin of mesenteric paraganglioma.^4^ Similar to the present case, it was reported that the small mesenteric paraganglia in the fatty tissue of the sigmoid mesocolon were accidentally resected together with the lymph nodes.^4^ In a different report in which the authors used formaldehyde‐induced fluorescence histochemical techniques, paraganglia fluorescent cell clusters were evenly distributed throughout loose abdominal connective tissues in an adult individual, indicating that the paraganglia persisted as a broad group after birth.^5^ Taking these reports into account, although small paraganglia exist in the mesentery, the small size and sparse number explain why paragangliomas are encountered so rarely.

In a different surgical case, a small paraganglia measuring around 1.0 mm in diameter at the root of the superior mesenteric artery was resected together with lymph nodes (Fig. S1u–x), and its cytological features closely resembled that of the mesenteric paraganglia in the present case. The small paraganglia was accidentally found microscopically, presumably not visible to the naked eye. Generally, paraganglia develop in the embryonic period and regress during the first decade of life. However, remnants can be found at any age in adult life as small paraganglia. The mesenteric paraganglia in the present case may not have regressed and remained macroscopically visible. Therefore, they were assumed to be lymph nodes during the surgical procedure of lymph node resection.

In the present report, we accidentally discovered and characterized a large mesenteric paraganglia as a lymph node of the sigmoid mesocolon initially. As far as we know, there is no report regarding mesenteric paraganglia as large as lymph nodes. Another important viewpoint of the present case is that when we encounter a large neuroendocrine tissue like in the present case among the lymph nodes, it should first be considered as paraganglia, not confused with lymph node metastasis of neuroendocrine tumors, especially carcinoid tumors.

## DISCLOSURE STATEMENT

None declared.

## AUTHOR CONTRIBUTIONS

Conception and design of the study: DS, SM and TI; data analysis: TK, YA, YE, HK, HY and IK; immunohistochemical analysis: SM, MS and SB; preparation of manuscript and figures: DS, SM and TI. All authors have read and approved the manuscript.

## Supporting information

Additional Supporting Information may be found in the online version of this article at the publisher's website.

Supporting information.

Supporting information.

Supporting information.
