# Supplementary material for: Incidentally discovered mesenteric paraganglia as large as a lymph node in the sigmoid mesocolon, a possible origin of mesenteric paraganglioma
Source: Pathol Int. 2020 Apr 27;70(7):476–8. doi: 10.1111/pin.12939 (PMC7384147; doi:10.1111/pin.12939)
Supplement: Supplementary file 2 — Supporting information. [file PIN-70-476-s002.docx]

**Supporting Figure 1**

**
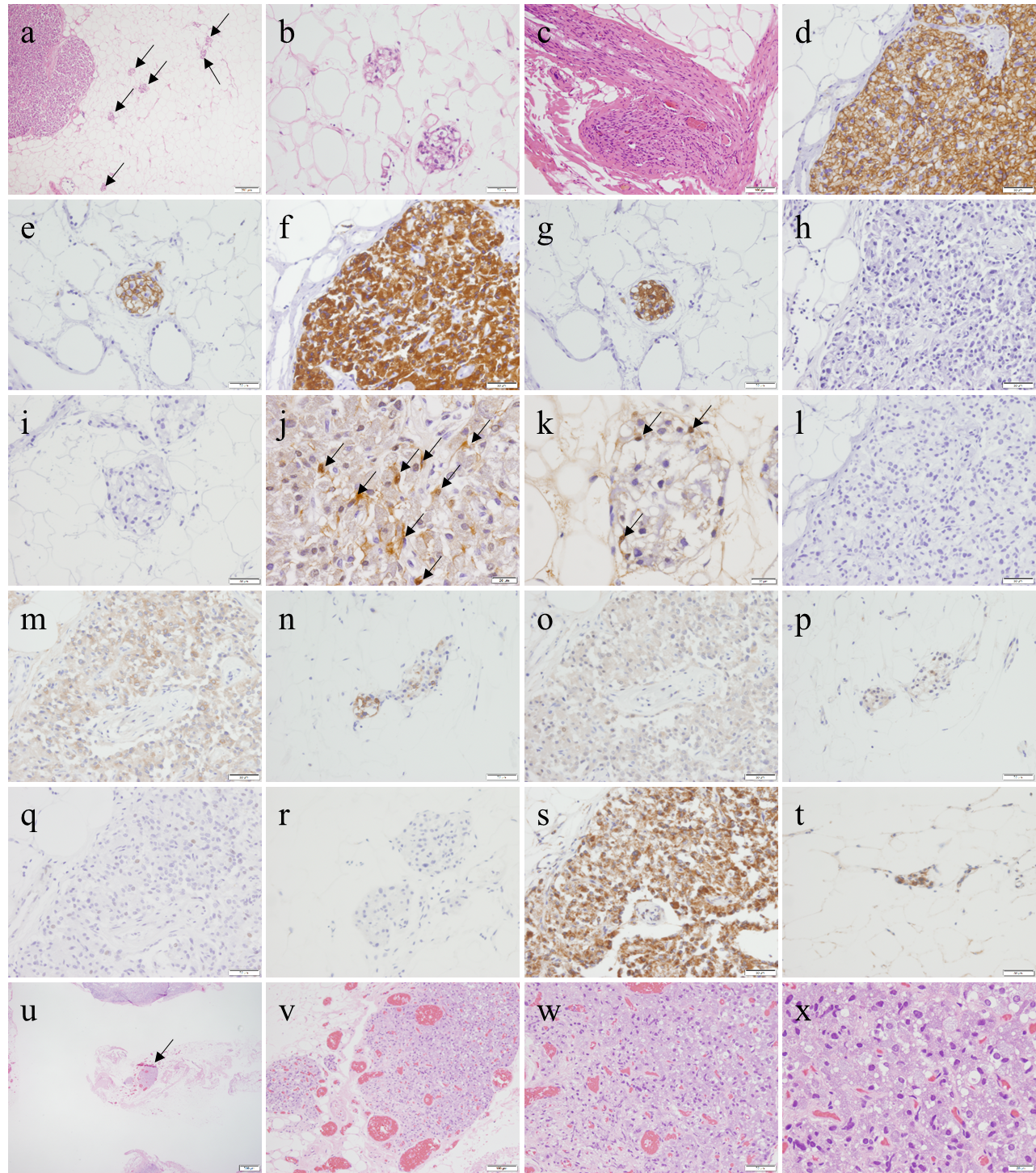
**

Figure S1. Lower (a) and higher (b) magnification image of the surrounding area of the nodules after hematoxylin and eosin staining (a:×40, b:×200). Arrows indicate the small nests around the nodules. (c) The myelinated peripheral nerves were found near the nodules (×100). The cells of the nodules (d, f, h, j, l, m, o, q, and s) and the tiny nests (e, g, i, k, n, p, r, and t) showed positive expressions of CD56 (d and e) and synaptophysin (f and g) (×200) and negative expressions of AE1/AE3 (h and i) (×200). Arrows indicate S100-positive sustentacular cells of the nodules (j) and the tiny nests (k) (×400). The Ki-67 labeling index of the tumor cells was less than 1% (×200) in the nodules (l). The cells of the nodules (m, o, q, and s) and the tiny nests (n, p, r, and t) showed positive expressions for tyrosine hydroxylase (m and n) (×200) and aromatic L-amino acid decarboxylase (o and p) (×200), but a negative expression for phenylalanine N-methyltransferase (q and r) (×200). The cells of the nodules (s) and the tiny nests (t) showed positive expression for SDHB (×200). A lower magnification image of the small paraganglia found in a different surgical case after hematoxylin and eosin staining (u) (×12.5). A higher magnification image of the small paraganglia after hematoxylin and eosin staining (v) (w) (x) (v:×100, w:×200, x:×400). Scale bars = 500 µm (u), 200 µm (a), 100 µm (c and v), 50 µm (b, d, e, f, g, h, i, l, m, n, o, p, q, r, s, t, and w), and 20 µm (j, k, and x).
