# Supplementary material for: Incidentally discovered mesenteric paraganglia as large as a lymph node in the sigmoid mesocolon, a possible origin of mesenteric paraganglioma
Source: Pathol Int. 2020 Apr 27;70(7):476–8. doi: 10.1111/pin.12939 (PMC7384147; doi:10.1111/pin.12939)
Supplement: Supplementary file 3 — Supporting information. [file PIN-70-476-s003.docx]

**Supporting References**

1. Asa SL, Ezzat S, Mete O. The diagnosis and clinical significance of paragangliomas in unusual locations. *J Clin Med* 2018; **7**: 280.

**Liver**

2. Liao W, Ding ZY, Zhang B, et al. [Primary functioning hepatic paraganglioma mimicking hepatocellular carcinoma: A case report and literature review.](https://www.ncbi.nlm.nih.gov/pubmed/29702975) *Medicine (Baltimore)* 2018; **97:** e0293.

**Orbit**

3. [Cesar M, Salinas-La Rosa](https://www.ncbi.nlm.nih.gov/pubmed/?term=Salinas-La%20Rosa%20CM%5BAuthor%5D&cauthor=true&cauthor_uid=27171205). Orbital Paraganglioma and Succinate Dehydrogenase Staining for Genetic Testing Triage and Prognosis. [*Ocul Oncol Pathol*](https://www.ncbi.nlm.nih.gov/pmc/articles/PMC4847686/) 2015; **2:** 36–9.

**Mandible**

4. [Sinha P](https://www.ncbi.nlm.nih.gov/pubmed/?term=Sinha%20P%5BAuthor%5D&cauthor=true&cauthor_uid=28670912), [Yuen SN](https://www.ncbi.nlm.nih.gov/pubmed/?term=Yuen%20SN%5BAuthor%5D&cauthor=true&cauthor_uid=28670912), [Chernock RD](https://www.ncbi.nlm.nih.gov/pubmed/?term=Chernock%20RD%5BAuthor%5D&cauthor=true&cauthor_uid=28670912), [Haughey BH](https://www.ncbi.nlm.nih.gov/pubmed/?term=Haughey%20BH%5BAuthor%5D&cauthor=true&cauthor_uid=28670912). Mandibular lytic lesion in familial paraganglioma syndrome type I: A clinical conundrum. [*Ann Otol Rhinol Laryngol*](https://www.ncbi.nlm.nih.gov/pubmed/?term=Mandibular+Lytic+Lesion+in+Familial+Paraganglioma+Syndrome+Type+I%3A+A+Clinical+Conundrum) 2017; **126:** 615–8.

**Paranasal sinus**

5. Nguyen BK, Patel NM, Arianpour K, et al. [Characteristics and management of sinonasal paragangliomas: a systematic review.](https://www.ncbi.nlm.nih.gov/pubmed/30570216) *Int Forum Allergy Rhinol* 2019; **9:** 413–26.

**Sellar region**

6. Ozüm U, Eğilmez R, Yildirim A. [Paraganglioma in pituitary fossa.](https://www.ncbi.nlm.nih.gov/pubmed/18410271) *Neuropathology* 2008; **28:** 547–50.

**Thyroid**

7. Lee S.M., Policarpio-Nicolas M.L. Thyroid Paraganglioma. *Arch Pathol Lab Med* 2015; **139:** 1062–7.

**Parathyoid**

8. [Michael TL](https://www.ncbi.nlm.nih.gov/pubmed/?term=Levy%20MT%5BAuthor%5D&cauthor=true&cauthor_uid=20237987), [John TB](https://www.ncbi.nlm.nih.gov/pubmed/?term=Braun%20JT%5BAuthor%5D&cauthor=true&cauthor_uid=20237987), [Marjorie P](https://www.ncbi.nlm.nih.gov/pubmed/?term=Pennant%20M%5BAuthor%5D&cauthor=true&cauthor_uid=20237987), [Lester DRT.](https://www.ncbi.nlm.nih.gov/pubmed/?term=Thompson%20LD%5BAuthor%5D&cauthor=true&cauthor_uid=20237987) Primary paraganglioma of the parathyroid: a case report and clinicopathologic review. [*Head Neck Pathol*](https://www.ncbi.nlm.nih.gov/pmc/articles/PMC2825535/) 2010; **4:** 37–43.

**Mediastinum**

9. [Yoshino N](https://www.ncbi.nlm.nih.gov/pubmed/?term=Yoshino%20N%5BAuthor%5D&cauthor=true&cauthor_uid=15141715), [Hisayoshi T](https://www.ncbi.nlm.nih.gov/pubmed/?term=Hisayoshi%20T%5BAuthor%5D&cauthor=true&cauthor_uid=15141715), [Maruyama Y](https://www.ncbi.nlm.nih.gov/pubmed/?term=Maruyama%20Y%5BAuthor%5D&cauthor=true&cauthor_uid=15141715), [Ogasawara H](https://www.ncbi.nlm.nih.gov/pubmed/?term=Ogasawara%20H%5BAuthor%5D&cauthor=true&cauthor_uid=15141715), [Yamauchi S](https://www.ncbi.nlm.nih.gov/pubmed/?term=Yamauchi%20S%5BAuthor%5D&cauthor=true&cauthor_uid=15141715), [Oaki Y](https://www.ncbi.nlm.nih.gov/pubmed/?term=Oaki%20Y%5BAuthor%5D&cauthor=true&cauthor_uid=15141715). Paraganglioma of the posterior mediastinum diagnosed by immunohistochemical staining. [*Jpn J Thorac Cardiovasc Surg*](https://www.ncbi.nlm.nih.gov/pubmed/15141715) 2004; **52:** 217–20.

**Lung**

10. Huang X, Liang QL, Jiang L, et al. Primary pulmonary paraganglioma: a case report and review of literature. *Medicine.* 2015; **94:** e1271.

**Heart**

11. Millar AC, Mete O, Cusimano RJ, et al. Functional cardiac paraganglioma associated with a rare SDHC mutation. *Endocr Pathol* 2014; **25:** 315–20.

**Gut**

12. Mete O, Pakbaz S, Cassol C, Asa S.L. The spectrum and Clinical Features of Paragangliomas. *Lab Investig* 2018; **98:** 234.

**Pancreas**

13. Meng L, Wang J, Fang SH. Primary pancreatic paraganglioma: A report of two cases and literature review. *World J Gastroenterol*. 2015; **21:** 1036–9.

**Mesentery**

14. Fujita T, Kamiya K, Takahashi Y, et al. Mesenteric paraganglioma: Report of a case. *World J Gastrointest* *Surg* 2013; **5:** 62–7.
